# The effect of benson relaxation technique on sleep quality and negative emotion in maintenance hemodialysis patients

**DOI:** 10.3389/fpsyt.2025.1662472

**Published:** 2026-01-14

**Authors:** Shan-Shan Wu, Jing-Fang Chen, Hong-Yun Yan, Tao-Feng Wu, Xian-Wen Li, Qin Gu, Jing Li, Ling Jiang, Xiao-Lan Fang

**Affiliations:** 1 Department of Nursing, Suzhou Municipal Hospital, The Affiliated Suzhou Hospital of Nanjing Medical University, Suzhou, China; 2 Hemodialysis Unit, Suzhou Municipal Hospital, The Affiliated Suzhou Hospital of Nanjing Medical University, Suzhou, China; 3 School of Nursing, Nanjing Medical University, Nanjing, China

**Keywords:** benson relaxation technique, maintenance hemodialysis, negative emotion, quality of life, sleep quality

## Abstract

**Objective:**

To explore the effect of Benson relaxation technique on sleep quality and negative emotion in maintenance hemodialysis patients.

**Method:**

A total of 105 patients with end-stage renal disease who underwent maintenance hemodialysis in the hemodialysis center of our hospital from September 1,2024 to April 1,2025 were selected as the research subjects. According to the order of enrollment, they were divided into study group (52 cases) and control group (53 cases). The scores of Pittsburgh sleep quality index (PSQI), self-rating depression scale (SDS), self-rating anxiety scale (SAS), 36-iem short form health survey (SF-36) and serum indexes [5-hydroxytryptamine (5-TH), interleukin-6 (IL-6), dopamine (DA)] before and after intervention were collected and compared.

**Result:**

After intervention, PSQI score (P<0.001), SAS score (P<0.001) and SDS score (P<0.001) in the study group were lower than those in the control group, and SF-36 score was higher than that in the control group (P<0.001). The scores of PSQI, SAS, SDS and SF-36 in the two groups were improved compared with those before the intervention. After intervention, DA in the study group (P<0.05) was lower than that in control group. There was no significant difference in the levels of IL-6 and 5-TH between the two groups after intervention (P>0.05).

**Conclusion:**

Benson relaxation technique can improve the sleep quality, anxiety and depression of patients with maintenance hemodialysis, and reduce the inflammatory response. It is an effective way to improve the prognosis and quality of life of patients with maintenance hemodialysis.

## Introduction

With the acceleration of population aging and the continuous change of lifestyle, the incidence of end-stage renal disease (ESKD) is gradually increasing. Maintenance hemodialysis (MHD) is the most widely used renal replacement therapy for patients with end-stage renal disease (1). However, compared with relatively healthy peers, MHD patients often face a significant reduction in functional ability. In addition, maintenance hemodialysis patients have long treatment time, high cost and many complications. The changes and adjustments of family roles and social adaptation will bring multiple pressures to patients, which is easy to produce changes in emotional and psychological state (2). This not only makes daily activities more challenging, but also increases the risk of various comorbidities and complications, which significantly affects their quality of life (3).

Sleep is a physiological activity necessary for the survival of living organisms. It is a recurrent physiological phenomenon in nature. The quality of sleep is closely related to environmental, physiological, psychological and behavioral factors. MHD patients are suffering from their own diseases and complications after dialysis. About 50% ~ 80% of patients have different degrees of sleep problems. Sleep disorders are manifested in different forms, such as easy awakening at night, extreme drowsiness during the day, fragmentation of sleep, dependence on sleeping drugs, etc. The results of Pojati´c et al. (4) showed that 41% -83% of MHD patients had sleep problems. Mirghaed M et al. (5) showed that the prevalence of sleep disorders in hemodialysis patients was 75.30%. Alshammari B et al. (6) found that the incidence of sleep disorders in MHD patients was as high as 89.7%. Long-term lack of sleep can easily lead to anxiety, depression and other negative emotions. Negative emotions can not only directly affect the patient’s sleep, but also indirectly affect the patient’s sleep quality by enhancing the inflammatory response and its secondary cardiopulmonary dysfunction (7). Anxiety and depression are significantly negatively correlated with sleep quality and quality of life in maintenance hemodialysis patients, which will increase the hospitalization rate of patients and lead to an increase in mortality (8).

In the treatment of hemodialysis patients, in addition to drug therapy, non-drug therapy as a complementary therapy or alternative therapy can also bring improvement in a short period of time. Psychological intervention, exercise training and relaxation therapy are commonly used non-drug treatment methods for dialysis patients (9). As a safe and low-cost non-pharmacological therapy, relaxation therapy can effectively alleviate patients’ negative emotions and stress states, improve patients’ treatment compliance, and promote patients to actively respond to diseases. It is one of the effective non-pharmacological treatment techniques (10).

Benson’s relaxation technique (BRT) is a relaxation therapy that can relieve bad mood, improve fatigue and sleep quality (11), and then improve the quality of life. It plays a great role in the field of nursing. Because of its less side effects, less invasive, easy to obtain, good curative effect and low cost, it is easy to be accepted by people. BRT is widely used in foreign countries, including patients with chronic diseases (12), surgical patients (13), cancer patients (14) and so on. However, there are few studies on BRT in China. At present, only a few studies are applied to hemodialysis and some cancer patients. The reason may be that there are many kinds of relaxation therapies in China, and they are more inclined to other relaxation therapies than BRT. Therefore, this has not been studied in depth. Moreover, few studies have explored the potential pathways by which BRT might influence neurochemical or inflammatory responses (e.g., dopamine, serotonin, or IL-6 levels) that are known to be associated with sleep quality and emotional regulation in hemodialysis patients: existing evidence from related relaxation−response interventions shows that BRT can increase dopamine and acetylcholine release (15), while mindfulness-based relaxation training (conceptually similar to BRT) has been demonstrated to reduce circulating interleukin−6 (IL−6) levels, a key inflammatory marker linked to sleep quality and emotional regulation (16).

Therefore, this study took maintenance hemodialysis (MHD) patients as the research subjects and discussed the influence of Benson Relaxation Therapy (BRT) on their negative emotions and sleep quality. By combining both psychological and physiological outcomes, this research addresses a significant gap in the existing literature. The study employs a more rigorous design, expanding the scope of relaxation therapy and enriching its content. This work provides valuable evidence for improving sleep quality, anxiety, and depression in patients with chronic diseases.

## 2 Materials and methods

### 2.1 Study subjects

The sample size was calculated by software, a total of 105 patients with end-stage renal disease who underwent maintenance hemodialysis in the hemodialysis center of our hospital from September 1, 2024, to April 1, 2025 were selected as the research subjects. Participants were randomly assigned to either the intervention group (Benson Relaxation Technique, n=52) or the control group (n=53) using a computer-generated randomization sequence at the time of enrollment. According to the order of enrollment, they were divided into a study group (52 cases) and a control group (53 cases). Inclusion criteria: (1) Age ≥ 18 years old; (2) Receiving maintenance hemodialysis for more than 3 months; (3) The patient was mentally normal, had no language communication disorder and was able to understand the purpose and significance of the investigation and volunteered to participate; (4) Diagnosed with mild sleep disturbance; (5) willing to participate. Exclusion criteria: (1) Patients who underwent MHD again after renal transplantation; (2) Those who were participating in similar clinical research; (3) Peritoneal dialysis at the same time; (4) daily life cannot take care of themselves; (5) Diagnosis of organic sleep disorders and patients receiving sleep therapy and taking sleep drugs. (6) Characteristics that disqualified someone (e.g., on sleep medication, severe psychiatric illness). Rejecting criteria: (1) Incomplete questionnaire data, data missing > 5%; (2) The questionnaire had obvious traces of regular answers.

This study was approved by the hospital ethics committee, ethics number K-2025-008-K01, patients signed informed consent.

### 2.2 Study method

The control group received routine nursing, including symptom management, dialysis care, medication guidance, and health education. The intervention group received the Benson relaxation technique in addition to routine nursing.

Before starting the intervention, the attending physician and the head nurse of the hemodialysis center were consulted to obtain consent and support. Nursing staff were informed of the study’s purpose to ensure cooperation and consistency in implementation. (1) Researchers introduced the concept, benefits, and procedures of the Benson relaxation technique, explaining its role in improving sleep and emotional well-being among hemodialysis patients. Educational sessions were provided to address knowledge gaps regarding relaxation therapy; (2) After obtaining informed consent, patients completed a general information questionnaire; (3) Researchers distributed instructional materials and audio guides for the Benson relaxation technique, trained patients on its correct practice, and established trust to enhance adherence. Patients were asked to demonstrate the relaxation process to confirm understanding; (4) Baseline data were collected for comparison before the intervention.

The implementation of the intervention in this study adopted a combination of group teaching and individualized guidance. During the relaxation process, the researchers guided the patients to wear headphones to listen to the audio. After the teaching, the subjects practiced relaxation under the supervision of the researchers to ensure that each intervention group was proficient in the therapy. (1) Relaxation process: ① Patients chose a comfortable lying position, handed on both sides of the body naturally, so that the body was in a comfortable state and position. The patient closed his eyes, wore an earphone provided by the researchers, adjusted the appropriate volume, isolated other murmurs in the ward, tried to eliminate the thoughts and follow the audio instructions to relax according to the steps. The audio simply guided the breathing, muscle relaxation and thinking imagination during the relaxation process. The patient could choose not to use the audio after being skilled. Patients need to consciously relax the body, adjust the psychological state, and feel the relaxation of the body muscles. The total order of relaxation of the body was from bottom to top, starting from the toes of both feet, followed by the ankle joint, two calves, knee joint, two thighs, hip joint, abdomen, chest, two arms, elbow joint, two arms, shoulder, neck, until the top of the head, focus on each relaxed part, relax and stretch each muscle group of each part in turn. ④ Adjusted the breathing, maintain the normal rhythm, tried to use slow deep breathing, paid attention to inhale with the nose, exhale with the mouth, slowly exhale and gently read ‘ 1 ‘ at the same time, can also silently read, to focus on, that was, inhale, exhale, and gently read ‘ 1 ‘ at the same time, repeat 20 minutes. ⑤ After the end, the patient slowly opened his eyes and kept in a supine position for 2–3 minutes before getting up or moving. (2) Operation plan: There was no clear standard and regulation on the duration and frequency of the intervention. In this study, through pre-experimental observation, combined with previous research experience, patients’ compliance and intervention effect, an 8-week intervention duration was adopted, 3 times a week, 20 minutes each time. After each completion, the Benson relaxation technique operation record sheet was filled out, and the record sheet was collected and checked by the researchers. (3) Noted patients should try to keep the surrounding environment quiet and comfortable, and the indoor light was soft. When the patient was not familiar with the process or the concentration was difficult to open the audio and wear headphones to follow the instructions, audio can be stored in various types of mobile phones and portable audio player; in the process of relaxation, patients should try to eliminate distractions, do not pay attention to the degree of relaxation, can open their eyes to check the time, but do not allow the use of alarm clock.

After each Benson relaxation technique, the subjects filled out the operation record sheet, filled out the relaxation time and feelings truthfully, and were collected by the researchers once a week. If missing items or inconsistent with the actual situation were found, the patients should be re-inquired and corrected. The researchers collected the patient’s telephone contact information in order to timely solve the patient’s questions during the operation, and established a WeChat group for regular supervision.

### 2.3 Quality control

The general data of patients, Pittsburgh sleep quality index (PSQI), self-rating depression scale (SDS), self-rating anxiety scale (SAS), 36-Item Short Form Health Survey(SF-36) score and serum data before and after intervention were collected: 5-hydroxytryptamine (5-TH), interleukin-6 (IL-6), dopamine (DA). 5-HT is a neurotransmitter critically involved in regulating sleep-wake cycles and emotional states, with research showing that low levels of 5-HT are associated with poor sleep quality and increased risk of depression (15). IL-6, as a pro-inflammatory cytokine, is linked to systemic inflammation, which has been found to affect sleep quality and contribute to depression and anxiety in chronic disease patients, including those undergoing maintenance hemodialysis (16).

The Pittsburgh Sleep Quality Index (PSQI) (17) was used to evaluate the quality of sleep. The scale included seven aspects: subjective sleep quality, sleep latency, sleep time, habitual sleep efficiency, sleep disorders, hypnotic drugs and daytime function. The total score was the sum of the scores of these seven aspects. The total score ranged from 0 to 21. The higher the score, the worse the sleep quality. 0 ~ 5: good sleep quality; 6 ~ 10: fair sleep quality; 11 ~ 15: general sleep quality; 16 ~ 21: Poor sleep quality.

The self-rating depression scale (SDS) (18) and the self-rating anxiety scale (SAS) (19) were used to evaluate the depression and anxiety of visitors. SDS score > 72 was severe depression; SDS score 63 ~ 72 was moderate depression; SDS score 53–62 was light depression; SDS score 69 points for severe anxiety need to be referred to professional psychological treatment institutions as soon as possible; the SAS score of 60 ~ 69 was moderate anxiety. The subjects often had some anxiety in the recent period of time, and they could generally adjust themselves. The SAS score of 50 ~ 59 was light anxiety. The subjects had occasional anxiety in the recent period of time, but their anxiety symptoms were light. After timely debugging, they would generally relieve quickly. SAS score < 50 points for no anxiety experience, belonged to the normal state.

As a concise health questionnaire, SF-36 comprehensively summarized the quality of life of respondents from eight aspects: physiological function, physiological function, physical pain, general health status, energy, social function, emotional function and mental health. The higher the score, the better the situation (20).

### 2.4 Statistical analysis

Statistical analysis was performed using SPSS Statistics software (version 26.0; IBM Corp., Armonk, NY, USA). The normality of the data was assessed using the Kolmogorov-Smirnov test. The measurement data that satisfied the normality assumption were expressed as mean ± standard deviation (x ± s). For comparisons between two groups, an independent t-test was used for between-group analysis, and a paired t-test was used for within-group comparisons before and after intervention. The indicators (IL-6, 5-HT, DA) that showed statistically significant differences in baseline data were analyzed using analysis of covariance (ANCOVA) to adjust for baseline differences. Confidence intervals (CIs) at 95% were reported for all significant differences. The count data were expressed as frequency (n) or rate (%), and differences between groups were compared using the chi-square (χ2) test for categorical variables that met the conditions. The Fisher’s exact test was applied when the expected frequencies were too low. A two-sided p-value < 0.05 was considered statistically significant. The sample size of each group was required to be more than 40 cases by using Gpower software to calculate (The statistical power were above 0.75). The cohen ‘d index was used to estimate the effect size of the t test. The effect size of this study were between 0.34 and 0.58.

## 3 Results

### 3.1 General data

During the study, 2 people in the study group were lost to follow-up, 1 person in the control group was lost to follow-up, and 2 people’s questionnaire data were incompletely filled out, so 50 people were included in each group for analysis. The results showed that there were 23 males and 27 females in the study group, with an average age of 56.20 ± 12.12 years. In the control group, there were 20 males and 30 females, with an average age of 57.18 ± 13.49 years. There was no significant difference in gender and age between the two groups (P > 0.05), as shown in **Table 1**.

**Table 1:** General data comparison.

Item	Study group(n=50)	Control group(n=50)	*P* value
Gender(Male/Female)	23/27	20/30	0.545
Age	56.20±12.12	57.18±13.49	0.703
Standard of culture			0.130
Primary school	7	5	
Junior high school / technical secondary school	30	39	
College or higher	13	6	
Marital status			0.611*
Wedlock	43	46	
Non-married	5	3	
Other	2	1	
Dialysis duration			1.000*
<12 months	3	2	
13–36 months	22	22	
37-84months	25	26	
History of renal surgery	6	4	0.505
Comorbidities	37	37	1.000
Experience related to relaxation therapy	4	2	0.678*

*Fisher's exact probability method was used.

### 3.2 Comparison of questionnaire indicators

The results showed that there was no significant difference in PSQI, SAS, SDS and SF-36 scores between the two groups before intervention, indicating that the patients were comparable (P > 0.05). After intervention, PSQI score (6.36 ± 0.94 VS 7.32 ± 0.79, P < 0.001), SAS score (62.20 ± 5.32 VS 67.50 ± 4.83, P < 0.001) and SDS score (51.22 ± 5.42 VS 57.44 ± 6.20, P < 0.001) in the study group were lower than those in the control group, and SF-36 score was higher than that in the control group (88.18 ± 5.12 VS 80.32 ± 4.85, P < 0.001). The PSQI, SAS, SDS and SF-36 scores of the two groups were improved compared with those before the intervention, as shown in **Table 2**.

**Table 2:** Comparison of questionnaire indicators.

Item	Time	Study group(n=50)	Control group(n=50)	*P* value	*95CI%*
PSQI	Before intervention	10.30±1.49	10.72±1.28	0.133	-0.971~0.131
	After intervention	6.36±0.94*	7.32±0.79*	<0.001	-1.306~-0.614
SAS	Before intervention	73.84±6.94	74.38±5.13	0.659	-2.961~1.881
	After intervention	62.20±5.32*	67.50±4.83*	<0.001	-7.316~-3.284
SDS	Before intervention	74.56±11.81	70.70±7.02	0.050	0.005~7.715
	After intervention	51.22±5.42*	57.44±6.20*	<0.001	-8.531~-3.909
SF-36	Before intervention	74.08±4.66	75.52±4.61	0.124	-3.280~0.400
	After intervention	88.18±5.12*	80.32±4.85*	<0.001	5.881~9.839

*The difference was statistically significant compared with the Before intervention.

### 3.3 Comparison of serum indicators

Due to the difference in baseline data of IL-6,5-TH and DA between the study group and the control group before intervention, the difference was statistically significant (P < 0.05). Therefore, covariance analysis was used to compare the IL-6, 5-TH and DA indexes of the two groups after intervention. The results showed that DA (44.54 ± 13.50 VS 65.67 ± 16.96, P < 0.001) in the study group was lower than that in the control group after intervention. There was no significant difference in IL-6 and 5-TH levels between the two groups after intervention (P > 0.05), as shown in **Table 3**.

**Table 3:** After intervention inflammation index comparison.

Item	Study group(n=50)	Control group(n=50)	*P* value	*95CI%*
IL-6	33.19±17.73*	41.08±16.32*	0.652	-5.392~4.229
5-TH	65.27±18.11*	77.37±20.73*	0.180	-6.316~2.315
DA	44.54±13.50*	65.67±16.96*	<0.001	-4.956~-1.11

*The difference was statistically significant compared with the Before intervention.

## 4 Discussion

Sleep disorders are one of the most common and serious symptoms in MHD patients. The causes and mechanisms of sleep disorders are very complex, and the exact mechanism is still unclear. There are relatively few interventions to improve sleep disorders in patients, and there is a lack of clinical guidelines for standardized diagnosis and treatment. Sleep disorders are manifested as insufficient sleep time and sleep deprivation. Insufficient nighttime sleep time can easily cause the cerebral cortex to enter a state of inhibition, manifested as dizziness, memory loss, inattention, and physical fatigue; sleep deprivation can easily lead to daytime fatigue, drowsiness and nighttime sleep disorders, which will not only reduce the quality of life, but also reduce the body’s immunity and cause endocrine dysfunction (21).

The results of this study showed that the improvement of sleep quality in the study group was higher than that in the control group after the intervention, which was consistent with the related research (22). The reason for the analysis may be: relaxation response training can effectively relieve the tension of the sympathetic nerve, can relax the muscles, reduce blood pressure and heart rate, regulate the patient’s physiological and psychological recovery, and promote the improvement of patients’ sleep quality (23). Benson relaxation technique can guide patients to passively ignore negative thoughts and emotions, reduce the stimulation of interference factors to sleep, and then improve the subjective sleep quality of patients (24). By improving sleep quality, promoting physiological rhythm to return to normal, alleviating patients’ fatigue, improving activity endurance, and improving the overall quality of life of hemodialysis patients. This findings are also in line with previous studies involving relaxation-based interventions in chronic conditions. A study on the effectiveness of Benson’s relaxation therapy in hemodialysis patients found significant improvements in sleep quality, including reduced sleep disturbances and better subjective sleep quality (25). Another study showed that relaxation techniques, such as mindfulness meditation and progressive muscle relaxation, can significantly improve sleep quality in patients with chronic diseases like kidney disease (26).

The comparison between the two groups after the intervention in the results of the study showed that there were statistically significant differences in the scores of SAS, SDS and SF-36 scales between the two groups, indicating that Benson relaxation technique can improve the anxiety and depression of maintenance hemodialysis patients. The therapy directly activates the relaxation response, thereby reducing physiological arousal and cognitive stress, which are key contributors to poor sleep and emotional distress. Sleep quality is closely related to anxiety and depression. Changes in physiological and biochemical aspects of the disease and factors such as fatigue, anxiety, worry, and depression play an important role in the pathogenesis of sleep disorders in patients. Sleep disorders also have a negative impact on the patient’s physiological, psychological, cognitive, and functional status (27). García-Llana et al. (28) found that depression was significantly associated with sleep disorders in patients. Patients with depressive tendencies were more likely to suffer from sleep disorders, and long-term sleep disorders would further cause negative emotions such as anxiety and depression. Therefore, effective relief of anxiety and depression can promote the improvement of sleep quality in maintenance hemodialysis patients. Hemodialysis patients experience long-term physiological and psychological stress, and improper handling of stress can reduce the quality of life, leading to physical, psychological, economic, social and emotional problems (29). Benson relaxation techniques can be used for self-regulation and management of stress, reduce stress levels, relieve negative emotions such as anxiety and depression, and improve quality of life and subjective well-being (30). However, no significant differences were observed in IL-6 and 5-HT levels after the intervention, which suggests that the effects of the relaxation technique may be more directly linked to mood, anxiety, and sleep quality rather than directly altering inflammatory or serotonin levels.

Long-term sleep disorders will seriously endanger human health and increase the probability of various diseases. Poor sleep quality can lead to disorders of physiological rhythms, decreased immunity, and changes in oxidative stress levels, resulting in increased mortality in MHD patients (31). Sleep has an important impact on the body’s various immune systems. Long-term sleep disorders affect the levels of immune cells such as lymphocytes, natural killer cells, immunoglobulins, and cytokines in the body. Compared with people with poor sleep quality, the number of lymphocytes in people with good sleep quality is significantly higher. Lymphocytes are the main force of the immune system. The ability of the human body to resist bacteria and viruses is closely related to the level of lymphocytes (32). Long-term sleep disorders make the body’s immune cells continue to decline, which in turn causes chronic, systemic inflammatory response, leading to inflammatory response-related diseases. Studies have found that microinflammation is common in MHD patients, which is closely related to renal failure, toxin accumulation, and decreased clearance of inflammatory cytokines (33).

In addition, the potential influence of the placebo effect and researcher involvement should be considered. Since the Benson relaxation technique is a psychological and behavioral intervention, participants’ awareness of being part of the intervention group might have contributed to perceived improvements in sleep and mood through expectancy or placebo effects. The active engagement and encouragement of researchers during the training and supervision process may also have enhanced patients’ motivation and emotional comfort, further influencing subjective outcomes such as anxiety and depression scores. Although these effects are difficult to eliminate completely in non-pharmacological studies, future research could minimize bias by including blinded assessors, using objective physiological indicators, or employing attention-control interventions to better isolate the specific effects of the relaxation technique itself.

Of course, this study also has some limitations. First, the included patients were all from a single hospital, which introduces regional and personnel limitations. There are problems such as a small sample size, short research time, and limited personnel. It is necessary to increase the number and scope of samples, extend the research time, and conduct deeper observations. In this study, polysomnography was not used to monitor the outcome indicators. In the future, more comprehensive, objective, and accurate physiological indicators and sleep parameters should be used for evaluation, such as polysomnography, electroencephalogram, eye movement map, etc., and more accurate monitoring of sleep staging changes. Additionally, the absence of long-term follow-up restricts conclusions about the sustainability of benefits. Future studies should consider longer follow-up periods to better assess the long-term effects of the intervention.

## 5 Conclusion

In summary, Benson relaxation technology can improve the sleep quality, anxiety and depression of maintenance hemodialysis patients, reduce the inflammatory response, and is an effective way to improve the prognosis and quality of life of maintenance hemodialysis patients.

## Data Availability

The original contributions presented in the study are included in the article/supplementary material. Further inquiries can be directed to the corresponding author/s.
